# Isolated renal sarcoidosis and concurrent secondary membranous nephropathy: a case report

**DOI:** 10.1186/s13256-025-05581-9

**Published:** 2025-10-23

**Authors:** Chandan Alenahalli Narayana, Faryal Safdar, Rachana Harish, Shunhua Guo, Ahsan Aslam

**Affiliations:** 1 Division of Nephrology and Hypertension, Indiana University School of Medicine, Indianapolis, Indiana United States; 2 Indiana University Fairbanks School of Public Health, Indianapolis, Indiana United States; 3 Department of Pathology, Indiana University, Indianapolis, Indiana United States

**Keywords:** Renal sarcoidosis, Membranous nephropathy, Interstitial nephritis, Autoimmune

## Abstract

**Background:**

Sarcoidosis is a chronic inflammatory disease characterized by abnormal T-cell responses to unknown antigens. It is a multisystem disorder, affecting the lungs and intrathoracic lymph nodes in over 90% of cases. Isolated renal sarcoidosis, a rare presentation, is confined to the kidneys without systemic involvement.

**Case presentation:**

A 46-year-old African American female was evaluated for persistently abnormal serum creatinine and pyuria. The only abnormality on her noninvasive workup was an elevated angiotensin converting enzyme level for which she was evaluated previously and she was not found to have any manifestations of sarcoidosis. We performed a kidney biopsy that showed findings of renal sarcoidosis and concomitant membranous nephropathy. Patient was treated with high-dose corticosteroids with stabilization of kidney function and resolution of sterile pyuria.

**Conclusion:**

Our case highlights that an elevated angiotensin converting enzyme level can predate the development of an overt renal sarcoidosis. It also shows that findings of a secondary membranous nephropathy can sometimes be seen in the absence of overt proteinuria in sarcoidosis involving the kidneys.

## Background

Sarcoidosis is a complex multisystem granulomatous disease with an uncertain etiology, primarily affecting the lungs and intrathoracic lymph nodes, though it can involve various organs [1]. Typically presenting in adults between 20 and 50 years old, sarcoidosis is driven by an abnormal T-cell response to unidentified antigens in genetically and environmentally predisposed individuals. This response leads to an increased presence of polarized macrophages and an imbalance between effector and regulatory T cells, resulting in the formation of non-caseating granulomas, commonly found in the affected organs [2]. While pulmonary involvement is most common, renal manifestations are rare, with clinically significant cases of renal sarcoidosis being even rarer [3]. Renal sarcoidosis is characterized by granulomatous inflammation within the kidneys, presenting a diagnostic challenge as it must be differentiated from granulomatous interstitial nephritis (GIN) caused by other factors such as drugs, infections, or autoimmune conditions [3, 4]. We present a case that reveals the rare combination of membrane nephropathy and granulomatous interstitial nephritis within isolated sarcoidosis, emphasizing the importance of considering sarcoidosis in the differential diagnosis of unexplained abnormal renal function.

## Case presentation

We present a case of a 46-year-old African American female with a medical history of hypertension, stage IV non-Hodgkin lymphoma in remission, and common variable immune deficiency (CVID). She was referred for concerns for worsening renal function. Twenty years ago, she experienced renal failure requiring 6 weeks of dialysis due to severe pneumonia but recovered and had normal renal function since then. She does not regularly use nonsteroidal anti-inflammatory drugs (NSAIDs) and denies any symptoms such as shortness of breath, lower extremity edema, or urinary issues. Her home medications include amlodipine, metoprolol, atorvastatin, fluticasone, and subcutaneous immunoglobulin (Ig) Hizentra 20% weekly for CVID. She has been following up regularly with her immunologist and has reported no recent infections. Regarding her lymphoma, she underwent chemotherapy and splenectomy about 5 years ago and has been in remission since, with regular follow-up with her hematologist. Her blood pressure is well-controlled on antihypertensives.

**Fig. 1:**
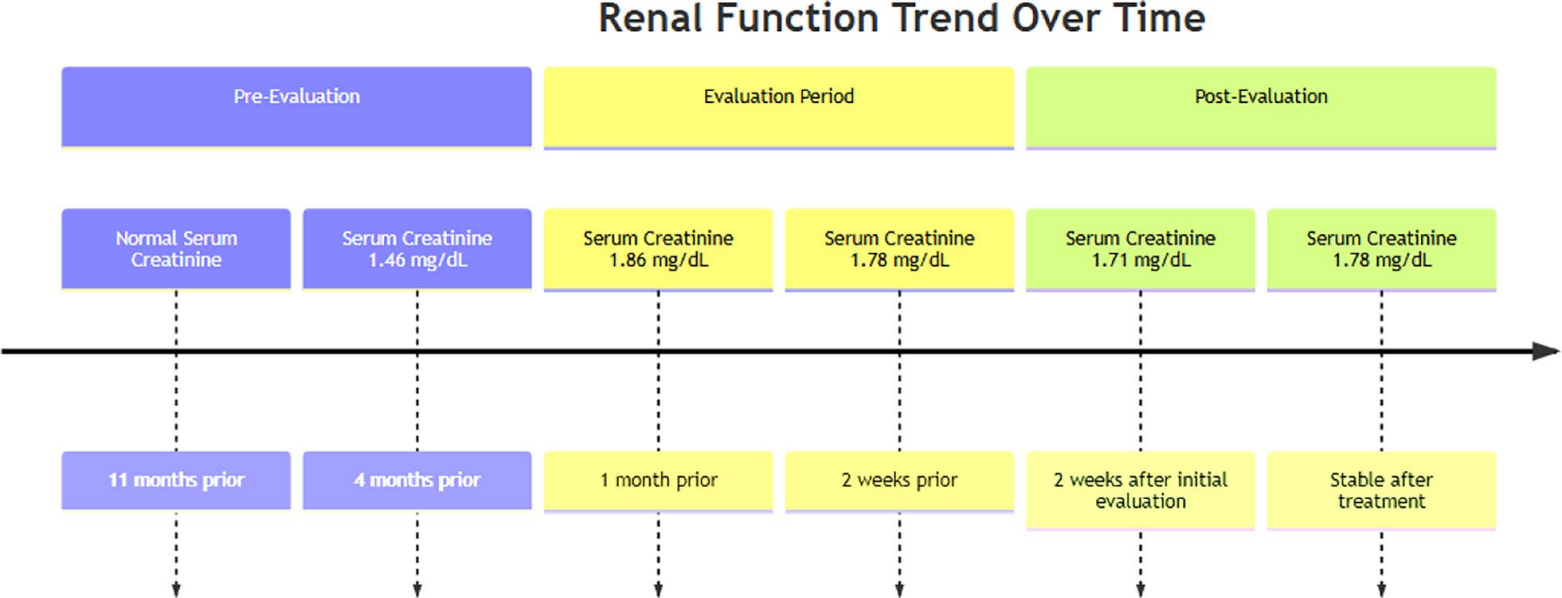
Timeline of kidney function trend

Serum creatinine was normal at 0.91 mg/dL with an estimated glomerular filtration rate (eGFR) of 79 about 11 months prior to nephrology evaluation. Four months before evaluation, as shown in Fig. 1, serum creatinine was 1.46 mg/dL with an eGFR of 45ml/min. Subsequent renal function values were: serum creatinine 1.86 mg/dL, eGFR 33 ml/min (1 month prior); creatinine 1.78 mg/dL, and eGFR 35ml/min (2 weeks prior). The patient was referred for evaluation of unexplained rising serum creatinine. Urinalysis showed trace leukocyte esterase and 11–20 white blood cells, with a negative eosinophil stain. Kidney ultrasound revealed kidney sizes of 11.9 cm and 12.3 cm for the right and left kidneys, respectively. Urine protein-to-creatinine ratio was 0.2 g/g, and microalbumin-to-creatinine ratio was 20 mg/g.

Differential diagnoses considered were tubulointerstitial nephritis versus osmotic nephropathy due to immunoglobulin use. Serum creatinine rechecked 2 weeks from the initial evaluation was 1.71 mg/dL. Urinalysis continued to show sterile pyuria. A kidney biopsy was performed to determine the precise cause of worsening creatinine and pyuria. The computed tomography (CT)-guided kidney biopsy was uneventful.

**Fig. 2:**
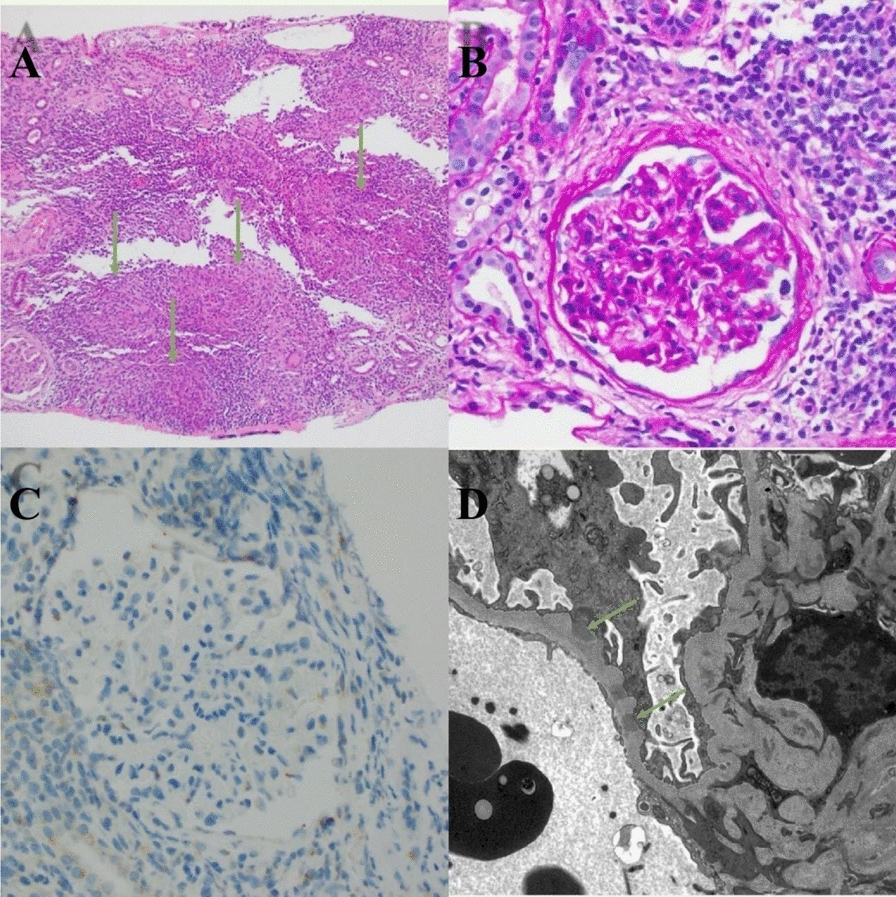
(**A**) Hematoxylin and eosin 100×: granulomatous inflammation foci (green arrows). (**B**) Periodic acid-Schiff (PAS) 400×: glomerulus without hypercellularity, crescents, or sclerosis. (**C**) Immunohistochemistry 100×/400×: stains were negative for cytomegalovirus (CMV), adenovirus, NELL1, EXT2, and SEMA3B. (**D**) Electron microscopy (EM) 2000×: subepithelial electron-dense deposits along the glomerular basement membrane (green arrows)

Light microscopy showed diffuse non-necrotizing granulomatous inflammation in the interstitium, moderate tubular atrophy, and interstitial fibrosis (30–40%). Immunofluorescence demonstrated diffuse granular glomerular capillary wall staining by IgG, C3, kappa, and lambda light chains. As shown in Fig. 2, no obvious spikes were noted by light microscopy, and electron microscopy showed only focal subepithelial electron-dense deposits, indicating an early stage of membranous nephropathy. Tissue was sent for PLA2R staining, which was negative, and serum PLA2R was also negative.

The differential diagnoses included drug exposure, infectious etiologies (for example tuberculosis, fungal infections, cytomegalovirus infection, and herpes simplex virus 1/2), herbal or other toxin exposure, and sarcoidosis. A thorough infectious workup was done: QuantiFERON was negative for tuberculosis, histoplasma antigen was negative, serum Epstein–Barr virus DNA was negative, and urine culture was negative. Her angiotensin-converting enzyme (ACE) level was mildly elevated at 77 units/L. About 9 months before nephrology referral, ACE was 112 units/L, with a stable 7 mm left lower lobe lung nodule and no thoracic lymphadenopathy noted at that time.

Diagnosis of renal sarcoidosis was made on the basis of elevated ACE levels and the biopsy findings. The patient was started on prednisone 60 mg daily, with *Pneumocystis jirovecii* prophylaxis (trimethoprim/sulfamethoxazole) and ulcer prophylaxis (omeprazole). Therapy was started given that patient had persistent pyuria and abnormal renal function. High-dose prednisone was continued for 2 months and repeat urine studies showed no white blood cells. Serum creatinine was stable at 1.78 mg/dL. The prednisone dose was reduced to 50 mg daily, with instructions to taper by 10 mg every 4 weeks until reaching 10 mg, then to continue at 10 mg daily. She was followed up closely in the renal clinic every 2–3 months to ensure stability of renal function and to determine any side effects of steroids. ACE levels were not rechecked since the urinary studies were found to be reassuring.

## Discussion

Sarcoidosis is a multisystem granulomatous illness with an unclear cause. The clinical hallmark of this condition is the presence of non-caseating granulomas in the affected organs, primarily the lungs and intrathoracic lymph nodes [5]. The majority of cases (80%) occur in individuals between the ages of 20 and 50 years. Patients typically present with one or more abnormalities, such as bilateral hilar adenopathy, pulmonary reticular opacities, and skin, joint, or eye diseases. Women and African American individuals are more likely to be affected [6].

Renal sarcoidosis, characterized by granulomatous inflammation confined to the kidneys, is an uncommon manifestation of systemic sarcoidosis. The incidence of renal involvement in sarcoidosis is approximately 30%, but it is usually clinically silent, with fewer than 5% of cases presenting with symptoms. The most prevalent histological entity is non-granulomatous tubulointerstitial nephritis (44%), followed by nephrocalcinosis (11%) and granulomatous interstitial nephritis (GIN, 30%). Other less common manifestations include proliferative or crescentic glomerulonephritis, membranous nephropathy, IgA nephropathy, minimal change disease, focal segmental glomerulosclerosis, renal masses, and urinary tract obstruction [7–10].

Diagnosing isolated renal sarcoidosis is challenging, as differentiating sarcoidosis-related GIN from GIN due to other causes can be difficult (see Table 1). Many studies have excluded GIN without extrarenal sarcoidosis to avoid misclassification of GIN caused by drugs, infections, or other autoimmune diseases [4, 11]. Some studies have relied on biomarkers such as elevated ACE levels, hypercalcemia, and response to steroids as diagnostic criteria for isolated renal sarcoidosis. However, the frequency of these biomarkers has been inconsistent [11–15]. In our case, the diagnosis of isolated renal sarcoidosis was based on elevated ACE levels combined with the biopsy findings, and the patient showed an excellent response to steroid treatment.

**Table 1:** Renal sarcoidosis versus other causes of granulomatous interstitial nephritis [3, 16]

Feature	Renal sarcoidosis	Other causes of granulomatous interstitial nephritis (GIN)
Etiology	Systemic sarcoidosis (idiopathic multisystem disease)	Infections (tuberculosis, fungal, and so on), medications (NSAIDs, antibiotics, PPIs), other systemic diseases (SLE, ANCA-associated vasculitis), idiopathic
Systemic Involvement	Often present (pulmonary, lymph nodes, skin, eyes, and so on)	May or may not be present, depending on the underlying cause
Hypercalcemia/hypercalciuria	Common (due to increased calcitriol production)	Less common, but can occur in some conditions (for example, tuberculosis, certain medications)
Renal manifestations	GIN is common; nephrocalcinosis, nephrolithiasis, rarely glomerulonephritis	GIN is the primary manifestation; other glomerular involvement depends on the underlying cause
Granuloma characteristics	Typically non-caseating	Can be caseating (for example, tuberculosis, fungal) or non-caseating
Clinical presentation	May be asymptomatic, or present with acute kidney injury (AKI), hypercalcemia-related symptoms, or features of systemic sarcoidosis	Variable, depending on the cause; can include AKI, fever, rash, arthralgias, and symptoms related to the underlying systemic disease
Associated findings	Elevated ACE levels, abnormal chest X-ray/CT, elevated serum calcium, high urine calcium	May have specific serological markers (for example, ANCA), positive cultures for infections, history of drug exposure
Renal biopsy	Non-caseating granulomas in the interstitium; may have tubular damage and interstitial fibrosis	Granulomas with or without necrosis (caseation); other features depending on the etiology (for example eosinophils in drug-induced GIN)
Treatment	Corticosteroids are the mainstay; immunosuppressants may be used in refractory cases	Treatment directed at the underlying cause (for example antibiotics for infections, withdrawal of offending drug); corticosteroids may be used to reduce inflammation
Prognosis	Generally good with treatment, but chronic kidney disease can occur in some cases	Variable, depending on the underlying cause and response to treatment

Multiple antigens, including PLA2R, NELL1, exostotin 2, PCDH7, SEMA3B, and THSD7A, have been described in association with sarcoidosis. However, no single target antigen accounts for all cases. The incidence of these target antigens in sarcoidosis appears to mirror the overall incidence of target antigens in membranous nephropathy (MN) [17]. Our patient tested negative for PLA2R, exostotin 2, and SEMA3B. We also encountered literature suggesting that underlying genetic susceptibility might explain the co-occurrence of these two diseases. The HLA-DRB1*0301 and HLA-DRB1*1501 genes have been associated with both membranous nephropathy and sarcoidosis [18–20]. It is postulated that membranous nephropathy in these patients is due to the expression of anti-PLA2R1 antibodies, which are thought to correlate with HLA-DRB1*0301.

The temporal relationship between membranous nephropathy and sarcoidosis varies widely. We came across a study of 33 patients with sarcoidosis who developed nephrotic syndrome; it was notable that 60% of these cases were membranous nephropathy. In ten cases, membranous nephropathy developed 8 years after sarcoidosis, while in four cases, it presented 10 years before sarcoidosis [21]. In another study, three cases presented with biopsy-proven membranous nephropathy and developed significant symptoms of sarcoidosis within a span of 3 months to 2 years [22]. Interestingly, serum ACE levels and serum calcium were normal in these cases, as they did not have any signs of sarcoidosis at the time of MN presentation.

In our patient, membranous nephropathy was identified by electron microscopy in the absence of proteinuria. There was no evidence of active malignancy (with lymphoma in remission) or any drug that could be causing it, strongly indicating membranous nephropathy secondary to sarcoidosis. High-dose prednisone led to the stabilization of renal function and the resolution of pyuria, consistent with previous reports of GIN responding well to corticosteroid therapy [12].

Another relevant aspect of our case was the history of non-Hodgkin lymphoma (NHL). Lymphoma can occur in patients with sarcoidosis and this relationship is due to dysfunction of the immune system that is present in these diseases [23]. Also, there have been reports of sarcoidosis development after chemotherapy for non-Hodgkin lymphoma was given [24]. Our patient had history of chemotherapy for NHL and sarcoidosis developed after that, leading us to consider whether the prior chemotherapy may have contributed to the development of sarcoidosis.

The treatment of renal sarcoidosis with corticosteroids has consistently shown good results. Newman *et al*. suggested that the optimal treatment for granulomatous nephritis is to start patients on 30–40 mg of prednisolone daily for 8–12 weeks, followed by a gradual tapering of the dose to 10–20 mg on alternate days over 6–12 months to establish the minimal effective dose [25]. Some papers also suggest a gradual taper over 2 years after the initial high dose to prevent relapse. If corticosteroids are ineffective or need to be spared, other treatments such as methotrexate or infliximab can be prescribed [26]. Corticosteroids reduce granulomatous inflammation and prevent irreversible fibrosis. The treatment of sarcoidosis is expected to prevent the development of proteinuria due to secondary MN since the underlying cause is being addressed. The long-term prognosis appears to be favorable in cases that are diagnosed and started on treatment earlier in the course of disease with steroids, such as our patient.

## Conclusion

This rare case of isolated renal sarcoidosis with both membranous nephropathy and granulomatous interstitial nephritis highlights the importance of considering sarcoidosis in a patient with unexplained renal dysfunction. Corticosteroids successfully stabilized renal function and prevented development of proteinuria due to the secondary MN. This case underscores the importance of a high index of suspicion for sarcoidosis in a patient with unexplained renal dysfunction with sterile pyuria. It also highlights that secondary MN can be seen in renal sarcoidosis, which, if noticed in early stages, can have no proteinuria.

## Data Availability

Data sharing is not applicable to this article as no datasets were generated or analyzed during the current study.

## Abbreviations

MN: Membranous nephropathy
ACE: Angiotensin converting enzyme
GIN: Granulomatous interstitial nephritis
PLA2R: Phospholipase A2 receptor
CT: Computerized tomography
CVID: Common variable immune deficiency
